# Magnetic solid-phase extraction technique based on Fe_3_O_4_@coPPy-PTH nanocomposite for extraction of cobalt, chromium, and nickel prior to determination by microsample injection system-flame atomic absorption spectrometry in alcoholic and nonalcoholic beverages

**DOI:** 10.55730/1300-0527.3683

**Published:** 2024-07-14

**Authors:** Melike KÜÇÜKSAKALLI, Qamar SALAMAT, Buket TİRELİ, Şükrü Gökhan ELÇİ

**Affiliations:** Department of Biomedical Engineering, Faculty of Technology, Pamukkale University, Denizli, Turkiye

**Keywords:** Fe_3_O_4_@coPPy-PTH nanocomposite, metal ions, microsample injection system-flame atomic absorption spectrometry, beer, wine

## Abstract

A novel Fe_3_O_4_@coPPy-PTH nanocomposite-based sorbent was prepared via in situ oxidative polymerization using Fe_3_O_4_ nanoparticles with spherical and flower-like morphologies of thiophene and pyrrole as the feedstocks. The synthesized nanocomposite displayed sensitive extraction and determination of metal ions Co(II), Cr(III), and Ni(II) without a chelating agent, followed by microsample injection system-flame atomic absorption spectrometry. Advanced spectroscopic and imaging techniques including scanning electron microscopy (SEM) and Fourier transform infrared spectroscopy were used to characterize the composition and morphology of the Fe_3_O_4_@coPPy-PTH nanocomposite. SEM observations showed that the size of the Fe_3_O_4_ nanoparticles changed from 30 nm to 120 nm in diameter after copolymer (PPy-PTH) coating. The Fe_3_O_4_@coPPy-PTH nanocomposite has good dispersion properties and stability in strong acid solutions. For effective extraction of the studied analytes, the influence of sample pH, volume of sample solution and eluent, amount of adsorbent, and interference of coexisting metal ions were optimized. Under the optimum conditions, preconcentration factors were obtained as 25 for all analytes. The calibration curves were linear in the range of 0.0–10.0 μg L^−1^ with coefficients of determination (R^2^) greater than 0.9957 for all three analytes. Limits of detection (S/N = 3) were calculated in the range of 0.17–0.23 μg L^−1^. Precision values, expressed as relative standard deviations, were lower than 3.0%, and relative recoveries were obtained in the range of 88.6%–103.6%. The proposed method (Fe_3_O_4_@coPPy-PTH/MSPE/MIS-FAAS) was successfully applied to extract and determine the studied metal ions in beer, wine, and nonalcoholic beverage samples.

## 1. Introduction

Heavy metals are commonly used in numerous industrial processes. However, their presence in industrial waste is considered a possible toxic pollutant to the environment. These metals may impact human health and be implicated directly or indirectly in food chains. Because of their nonbiodegradable nature, lengthy biological half-life, and ability to accumulate inside living beings, many trace elements are toxic even at very low doses [1]. The vitamin B_12_ active sites are constructed by cobalt, an important trace metal for the health of humans and animals. Elevated levels of cobalt inside the human body have been associated with several adverse health effects, including thyroid dysfunction, cardiovascular disorders, gastrointestinal disturbances such as diarrhea and pain, and impaired enzyme activity, as well as symptoms of nausea. Nickel ions interact with nucleic acids to form RNA and DNA [2]. Anemia, osteoporosis, cancer, severe allergies, and respiratory issues are a few health impacts of nickel [3]. The correct metabolism of glucose, protein, and fat depends on chromium(III), which makes it a crucial dietary component. Chromium(III) overdose can harm the kidneys or liver and cause digestive issues [4]. For nickel, cobalt, and chromium(III), daily intakes of 50–200 μg, 5.0–50 μg, and 50–200 μg, respectively, are advised [5].

The monitoring of metal ion levels, which include cobalt, chromium, and nickel, is crucial due to their significant influence on product quality, environmental conservation, and health safety. Although certain metals are necessary in small quantities, higher concentrations of certain metals can lead to severe health issues such as allergies, cancer, and damage to organs. The chemical amounts are checked often to make sure they remain below the allowed limits. This protects consumers from any possible harmful effects [6]. These metals can also change the look and taste of drinks. If there are too many of these substances in a product, it can change the color and taste, which lowers the product’s value. To avoid problems with the law and keep customers’ trust, it is important to follow the strict rules set by regulatory bodies regarding the legal levels of certain metals in consumables. If the source or type of the metal is known, it is easier to determine the source of the contamination, such as processing tools, raw materials, or packaging, and fix the problem. Beverage companies that address cobalt, chromium, and nickel levels will protect public health, keep product quality high, meet government requirements, and promote sustainable development [6]. It can thus be inferred that identifying cobalt, nickel, and chromium in trace amounts in different matrices is crucial in several fields, including environmental science and food chemistry.

A variety of analytical techniques have been devised for the detection and characterization of metals. These approaches include inductively coupled plasma-optical emission spectrometry (ICP-OES), inductively coupled plasma mass spectrometry, ultraviolet-visible spectrometry (UV-Vis), and flame atomic absorption spectroscopy (FAAS) [7–9]. The direct quantification of trace metal contents in complex matrices, such as environmental samples, is currently constrained due to the intricate nature of the matrices and the normally low amounts of metals involved. These limitations result in measurements that often approach or fall below the detection limits of the employed techniques. These restrictions are easily overcome by applying efficient sample preparation techniques before the analysis with instruments that enable the examination of complicated samples and the identification of analytes at minimal concentrations. These aims can be achieved by employing extraction techniques that include transferring the analyte from the original solution to a second phase that is compatible with the analytical tool being utilized. In recent years, various advanced techniques have replaced traditional extraction methods. These approaches provide several benefits, including reduced time requirements, improved extraction efficiency, minimal chemical usage, and cost-effectiveness. These novel strategies mostly rely on the reduction in size of uncomplicated extraction methods. Microextraction approaches, also known as miniaturized layouts, were developed to overcome the limits of classic extraction procedures while retaining their advantages. Microextraction techniques are highly regarded due to their ability to selectively and sensitively enrich and concentrate specific analytes from complicated matrices using microscale technologies. They are economically advantageous and environmentally friendly due to the limited use of solvent. Various forms of microextraction rely on different extraction techniques for analyzing food samples. Examples of such approaches include micro solid-phase extraction (μSPE) [10] and liquid-phase microextraction (LPME) [11].

μSPE is widely regarded as the preferred method due to the technique’s inherent simplicity, rapidity, capacity to achieve substantial concentration factors, and potential for reusing the adsorbent material. Additionally, μSPE is favored for its cost-effectiveness, as well as its minimal reliance on organic solvents [12]. Recently, the development of nanoscale adsorbents for the SPE process has been the focus of numerous research projects. Nanoparticles (NPs) have a much larger surface area-to-volume ratio than micrometer-sized particles and a shorter diffusion path. This leads to high extraction capacity, quick extraction dynamics, and high efficiency [13]. The efficacy and applicability of magnetic nanoparticles (MNPs) as SPE adsorbents for the enrichment of minute amounts of organic and inorganic analytes are the subject of current investigation [14].

In the context of magnetic solid-phase extraction (MSPE), the sample solution is subjected to the dispersion of the magnetic nanosorbent, facilitating the attainment of phase separation through the application of an external magnetic field positioned external to the sample container. However, the material does not exhibit residual magnetism with the removal of the magnetic field [14]. It is noteworthy that naked MNPs tend to aggregate, forming substantial clusters. This phenomenon has the potential to alter the magnetic properties of the constituent particles. Additionally, these nanometer-sized sorbents are not targeted due to their low capacity and are inappropriate for samples with complex matrices [15–19]. The enhancement of dispersibility and provision of an active surface for targeted molecular interactions can be achieved by surface modification.

In the present study, a coating of polypyrrole (PPy) and polythiophene (PTH) copolymer nanocomposite on the surface of Fe_3_O_4_ NPs (Fe_3_O_4_@coPPy-PTH) was used as a nanosorbent for the preconcentration of cobalt, chromium, and nickel ions at trace levels in the MSPE technique before their determination using microsample flame atomic absorption spectroscopy (MIS-FAAS). The utilization of the MSPE-MIS-FAAS technique constitutes a promising approach that is both cost-effective and environmentally sustainable for the quantification of the studied analytes in diverse beer and wine samples.

## 2. Materials and methods

### 2.1. Materials and reagents

All reagents used were of analytical grade. Ferric chloride anhydrous (FeCl_3_) and ferrous chloride tetrahydrate (FeCl_2_·4H_2_O) were obtained from Sigma-Aldrich (St. Louis, MO, USA). Ammonia solution (25%; NH_3_), thiourea, hydrochloric acid (37%; HCl), nitric acid (65%; HNO_3_), hydrogen peroxide (H_2_O_2_), and sodium hydroxide (NaOH) were all purchased from Merck (Darmstadt, Germany). Synthetic-grade thiophene and pyrrole were obtained from Merck. Standard stock solutions (1.000 mg L^−1^) of cobalt, chromium, and nickel were prepared by dissolving appropriate amounts of analytical grade Co(NO_3_)_2_, Cr(NO_3_)_3_, and Ni(NO_3_)_2_ salts from Merck in ultrapure water, respectively. The preparation of mixed working standard solutions involved the dilution of stock solutions with ultrapure water.

pH modifications were performed utilizing several buffer solutions. The H_2_PO_4_^−^/H_3_PO_4_ buffer was employed to achieve a pH of 2, while CH_3_COO^−^/CH_3_COOH buffers were utilized to get pH values ranging from 4 to 6. H_2_PO_4_^−^/HPO_4_^−2^ buffers were employed to alter the pH within the range of 6.5 to 7.5, and NH_4_^+^/NH_3_ buffers were used to achieve pH values ranging from 8 to 11. High-purity water with resistance of 18.2 MΩ cm^−1^ was generated using a reverse osmosis system manufactured by Human Corporation in Seoul, Korea.

The accuracy of the procedure was assessed using the certified reference material SPS-WW2, which contains metal ions found in industrial effluents. Authentic specimens were obtained from a domestic marketplace located in Denizli, Türkiye.

### 2.2. Instrumentation and apparatus

The flame atomic absorption spectrometer utilized in this study was a PerkinElmer AAnalyst 200 (PerkinElmer, Norwalk, CT, USA). It was outfitted with hollow cathode lamps containing chromium, nickel, and cobalt. The instrument was equipped with a manually fabricated microsample injection system (MIS) to enable the analysis of smaller sample volumes while maintaining high levels of accuracy and reproducibility with a strong absorbance signal [20]. The instrumental parameters utilized for the determination of chromium, nickel, and cobalt were established by the manufacturer’s handbook guidelines. The utilization of MIS facilitated the acquisition of a high-quality signal by employing a sample volume of 100 μL, which could be conveniently injected into the instrument’s nebulizer using a micropipette [21]. The experimental setup involved the utilization of the pH720 model pH meter (WTW, Weilheim, Germany), a heating magnetic stirrer (Velp Scientifica ARE, Usmate, Italy), and an ultrasonic bath (Ultrasound Bendelin Electronics, Berlin, Germany). An ATR-IR spectrometer, namely the PerkinElmer Spectrum Two with the UATR accessory (Norwalk, CT, USA), was utilized to acquire ATR spectra. The spectrometer was equipped with a germanium crystal and a Fourier transform infrared (FTIR) microscope attachment. The NPs were subjected to scanning electron microscopy (SEM) and energy-dispersive X-ray spectroscopy (EDS) analysis using a Supra 40VP apparatus (Zeiss, Jena, Germany) at the Application of Advanced Technology and Research Center of Pamukkale University. The reverse osmosis system manufactured by Human Corp. (Seoul, Korea) was employed to acquire water of ultrapure quality, characterized by resistance of 18.2 MΩ cm^−1^.

### 2.3. Synthesis of Fe_3_O_4_@coPPy-PTH

Initially, the Fe_3_O_4_ NPs were generated using a modified chemical coprecipitation method as described in a prior investigation [20]. A mixture of FeCl_2_.4H_2_O (1.43 g) and anhydrous FeCl_3_ (2.34 g) was homogeneously distributed in 70 mL of ultrapure water under continuous mechanical agitation. Upon reaching a temperature of 85 °C, 5 mL of ammonia solution (25 wt.%) was promptly introduced and the reaction was thereafter allowed to proceed for an additional 5 min. Subsequently, the black product, specifically iron(II, III) oxide (Fe_3_O_4_), was retrieved utilizing an external magnet, while the liquid portion above it was separated by the process of magnetic decantation. The produced Fe_3_O_4_ NPs were washed with deionized water multiple times and subsequently baked in a vacuum oven at a temperature of 80 °C for 10 h. Following that, to achieve the Fe_3_O_4_@coPPy-PTH nanocomposite, 0.5 g of the dried Fe_3_O_4_ NPs was added to 80 mL of deionized water and ultrasonically stirred for 15 min. Anhydrous FeCl_3_ (5.8 g) was added to the mixture as a catalyzing agent. In the next step, a solution containing 0.750 mL of pyrrole and 0.750 mL of thiophene was added dropwise under stirring. The mixture was then held for 8 h at room temperature. Finally, Fe_3_O_4_@coPPy-PTH was filtered and placed in an oven at 80 °C for 10 h to dry completely.

### 2.4. Characterization of Fe_3_O_4_@coPPy-PTH

**Figure 1:**
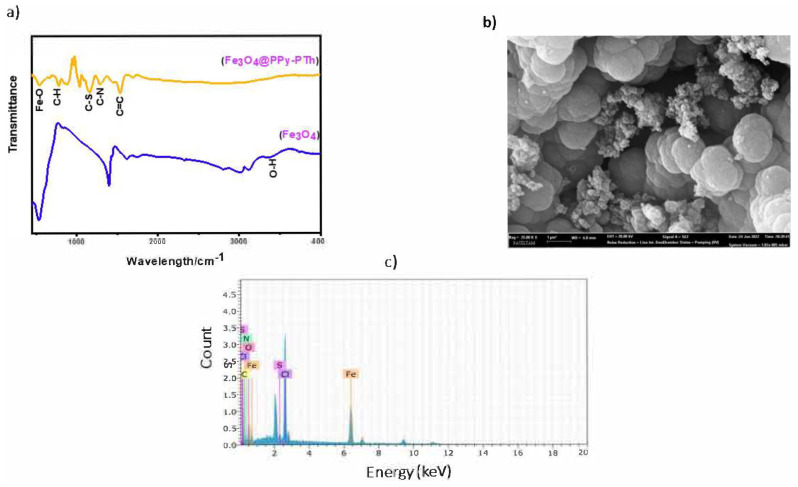
a) FTIR spectrum of Fe_3_O_4_ nanoparticles and Fe_3_O_4_@coPPy-PTH nanocomposite, b) scanning electron microscopic images of Fe_3_O_4_@coPPy-PTH, c) energy dispersive X-ray spectra of Fe_3_O_4_@coPPy-PTH.

The product’s structure was characterized with the PerkinElmer Spectrum Two FTIR instrument in the range of 500–4000 cm^−1^ to confirm the synthesis of the sorbent. The following results of the comparison between Fe_3_O_4_@coPPy-PTH and bare Fe_3_O_4_ nanomaterials are shown in Figure 1a: The observed peak at about 1543 cm^−1^ can be attributed to the stretching vibration of the carbon-carbon (C=C) double bond. The absorption at 1306 cm^−1^ is assigned to the C-N ring stretching band of pyrrole. The peaks at about 1169 cm^−1^ for the nanocomposite belong to the C-S stretching vibration in the thiophene ring [22]. The characteristic stretching vibration peak at 547 cm^−1^ in the Fe-O bond is associated with the magnetic phase. The weak peak at 1700 cm^−1^ is associated with the C-C ring stretching of pyrrole [23,24]. The peak at 789 cm^−1^ is caused by the vibration of C-H, which is characteristic of the C-C linkage in the thiophene ring [25]. The peak observed at a wavenumber of 547 cm^−1^ is indicative of the characteristic peak commonly associated with Fe_3_O_4_ [26]. The full synthesis of the Fe_3_O_4_@coPPy-PTH nanocomposite was validated using FTIR analysis.

The size and morphology of Fe_3_O_4_@coPPy-PTH nanocomposites and bare Fe_3_O_4_ were investigated with SEM imaging. According to the results, growth in particle size was observed from 30–35 nm for Fe_3_O_4_ to 100–120 for Fe_3_O_4_@coPPy-PTH (Figure 1b), indicating the copolymer coating on the surface of the Fe_3_O_4_ NPs. Furthermore, the stoichiometry of the materials was observed using EDS in conjunction with the SEM images. Figure 1c demonstrates the EDS spectra of Fe_3_O_4_@coPPy-PTH.

### 2.5. Extraction procedure

**Figure 2:**
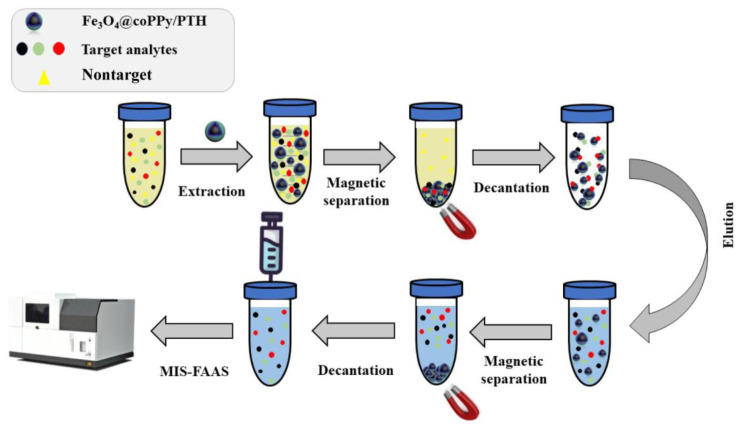
Schematic of the Fe_3_O_4_@coPPy-PTH/MSPE/MIS-FAAS procedure for metal ion extraction and determination.

Extraction was carried out with the following steps: Initially, a volume of 25 mL of the sample solution, which had been intentionally contaminated with a concentration of 100 μg mL^−1^ of Co(II), Cr(III), and Ni(II), was carefully transferred to a beaker. The pH of the sample solution was subsequently modified to 8.0 through the utilization of an ammonia buffer solution. Subsequently, a precise measurement was taken of 50 mg of Fe_3_O_4_@coPPy-PTH nanocomposites, which were then introduced into the solution as an adsorbent. Subsequently, shaking by hand was applied for 5.0 min to achieve a comprehensive dispersion of the nanocomposites within the solution, as well as to facilitate the adsorption of the analytes onto the surface of said nanocomposites. Afterward, the adsorbed analytes were collected from the resulting solution with an external magnet and then the supernatant was decanted. Subsequently, the analytes that had been adsorbed onto the adsorbent surface were desorbed by employing a solution consisting of 1.0 mL containing 0.2% thiourea in a mixture of 2.0 mol L^−1^ HCl and 1.0 mol L^−1^ HNO_3_. After 15 min of sonication and 10 min of centrifugation at 10,000 rpm, the supernatant was collected with a magnet. A volume of 750 μL of the extracted analyte was subsequently introduced into the MIS-FAAS system. The extraction technique is depicted in Figure 2.

### 2.6. Real sample preparation

The detection of analytes Co(II), Cr(III), and Ni(II) was assessed in selected beverages (beer, red wine, rosé wine, and white wine) by MIS-FAAS analysis. All beverage samples were purchased from a local market in Denizli, Türkiye. To achieve the thorough decomposition and removal of the organic matter from the samples under investigation, a wet-acid digestion method was employed, utilizing open-vessel conditions. The decomposition procedure was performed for all samples according to the literature [27] with a minor alteration. In summary, an aliquot of wine samples (100 mL) was transferred to a 250-mL Erlenmeyer flask and then 12 mL of concentrated HNO_3_ and 12 mL of concentrated H_2_O_2_ were added. The mixture was subjected to gentle heating. The heating process was prolonged until the volume of the sample was significantly reduced, approaching a state of near dryness. The volume of the obtained solution was completed to 25 mL with deionized water. The subsequent phase involved the implementation of the extraction protocol, utilizing the most favorable conditions as elucidated in Section 2.5. The beer sample was prepared with the same procedure without adding the concentrated H_2_O_2_ in the first step. Finally, the extracted analytes were determined by MIS-FAAS.

### 2.7. Statistical evaluation

For the statistical comparison of the data obtained, three replicate measurements were performed and all analytical parameters were calculated for method optimization and validation.

## 3. Results and discussion

To achieve optimal experimental conditions, parameters including pH, eluent volume, sample volume, and Fe_3_O_4_@coPPy-PTH quantity were tuned utilizing a one-variable-at-a-time technique. The experiments were performed in triplicate and averaged values were used as analytical signals. The findings are assessed and analyzed in the subsequent sections.

### 3.1. Effect of pH

**Figure 3:**
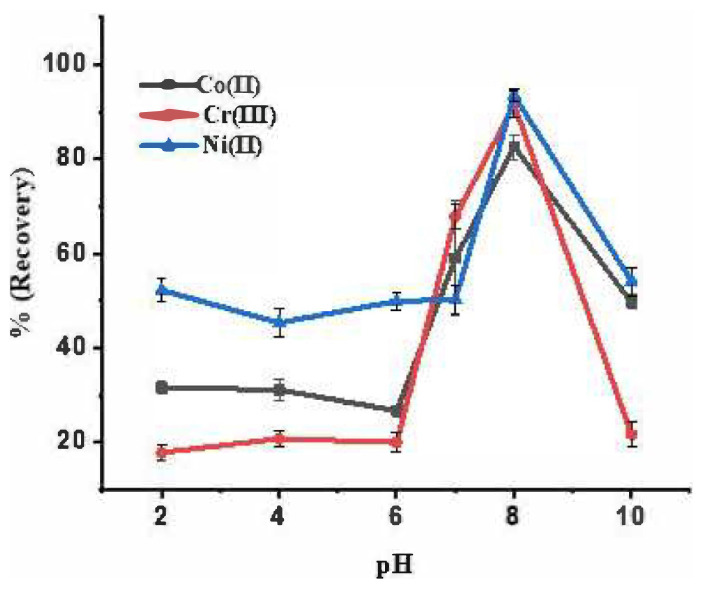
Effect of pH on the extraction recovery of metal ions. Extraction conditions: sample volume, 25 mL; eluent volume, 1 mL; absorbent amount, 50 mg; analyte concentration in sample solution, 10 μg L^−1^.

The pH of the sample solution is the critical factor in the extraction process of target analytes. This is because it influences the adsorption of the ions, which is influenced by both the nature of the metal ions inside the solution and the protonation of the donors present on the adsorbent. Hence, an assessment was conducted to determine the extraction effectiveness of the analyzed analytes (Co(II), Cr(III), and Ni(II)) on the surface of the adsorbent concerning the pH of the sample solution within the range of 2.0 to 10. In very acidic solutions, reduction in extraction effectiveness can be related to the donor protonation on the adsorbent, namely the nitrogen (−N) and sulfur (−S) atoms. This protonation leads to the occupation of active sites on the PPy-PTH copolymer by protons instead of the desired metal ions. Furthermore, in strong alkaline solutions (pH of more than 8), a decrease occurred in the extraction efficiency, probably due to the formation and precipitation of hydroxide species of the target metal ions. Based on the findings depicted in Figure 3, a pH value of 8 was chosen for the following studies to optimize the extraction efficiency.

### 3.2. Effect of sample solution and eluent volume

**Figure 4:**
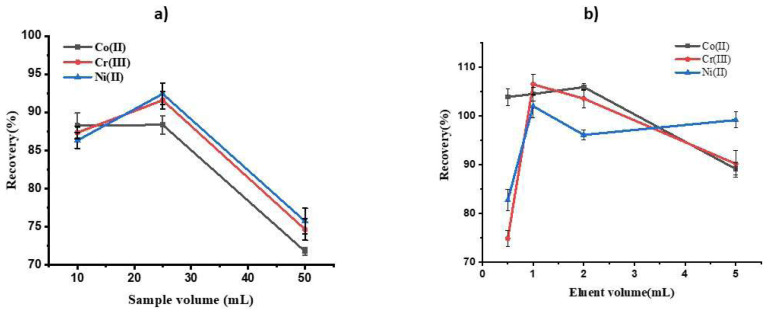
a) Effect of sample volume on the extraction recovery of metal ions. Extraction conditions: pH, 8; eluent volume, 1 mL; absorbent amount, 50 mg; analyte concentration in sample solution, 10 μg L^−1^. b) Effect of eluent volume on the extraction recovery of metal ions. Extraction conditions: pH, 8; sample volume, 25 mL; absorbent amount, 50 mg; analyte concentration in sample solution, 10 μg L^−1^.

The preconcentration factor (PF) is another important variable demonstrating a proposed method’s efficiency. To obtain the optimal PF, two parameters should be checked: the initial sample solution volume and the volume of eluent. Due to low metal ion concentrations being present in the initial sample solutions, a higher PF should be achieved. Experiments were performed for 10, 25, and 50 mL of sample solutions containing 100 μg L^−1^ of each analyte to understand the effect of sample volume. Figure 4a shows that the highest recovery was achieved with 25 mL of initial sample volume. The decrease in recovery observed with greater sample quantities can be attributed to a reduction in the interaction between target analytes and sorbent material.

Regarding eluent volume, various volumes in the range of 0.5–5.0 mL of a mixture composed of thiourea (0.2%), HNO_3_ (1.0 M), and HCl (2.0 M) were used as the eluent to desorb the analytes from the surface of the adsorbent. As shown in Figure 4b, 1.0 mL of the eluent obtained the highest extraction recovery. Therefore, these optimum sample solutions and eluent volume were applied in further experiments. Using the obtained optimal values, a PF of 25 was obtained.

### 3.3. Effect of Fe_3_O_4_@coPPy-PTH amount

**Figure 5:**
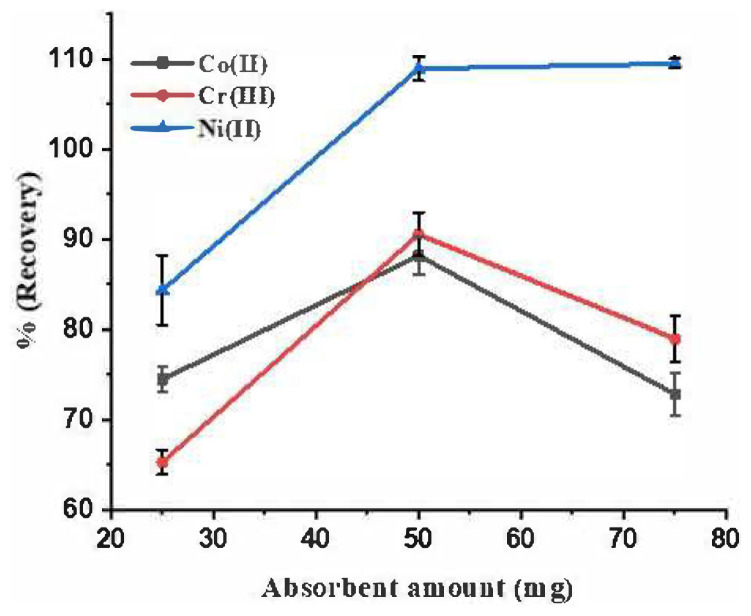
Effect of absorbent amount on the extraction recovery of metal ions. Extraction conditions: pH, 8; sample volume, 25 mL; eluent volume, 1 mL; analyte concentration in sample solution, 10 μg L^−1^.

The amount of Fe_3_O_4_@coPPy-PTH is one of the determining factors in extraction recovery through the adsorption of the studied analytes from the bulk of the solution. Hence, the effect of various amounts of solid nanocomposite (25, 50, and 75 mg) was investigated. According to the results shown in Figure 5, the maximum recovery was observed for 50 mg of Fe_3_O_4_@coPPy-PTH. At less than 50 mg, the nanocomposite amount was insufficient to adequately adsorb analytes. On the other hand, the decrease in recovery with an amount higher than 50 mg was due to inadequate eluent volume. Thus, 50 mg was used as an optimum amount of absorbent for further experiments.

### 3.4. Effect of interfering metal ions

The observed variation in the quantification of examined metal ions in real samples can be attributed to the matrix effect, wherein the interference of other metal ions plays a significant role. Despite the intricate composition of the matrices found in the water samples, the influence of foreign ions exhibited a level of similarity to our prior investigation with a comparable solid-phase substance [20].

### 3.5. Method validation

The validation of the method is crucial for proving the correctness of a developed novel method. In this work, after achieving optimum conditions for Fe_3_O_4_@coPPy-PTH-MSPE, the method was evaluated in terms of the limits of detection (LODs), linear dynamic range (LDR), coefficient of determination (R^2^), intraday and interday precision (RSD), and PFs. The parameters under investigation were examined through the addition of standard solutions to wine samples at 10 distinct concentrations. The findings are presented in Table 1. The LOD was determined based on a signal-to-noise ratio of 3:1 (S/N = 3), with a range of 0.17–0.23 μg L^−1^. The coefficient of determination (R^2^) ranged from 0.978 to 0.9992. The level of precision exhibited by donation was within the range of 1.2%–3.0% as measured by the relative standard deviation (RSD). All experiments were conducted in triplicate using water as a solvent. Furthermore, an assessment was conducted to evaluate the precision of the created technique by replicating the identical experimental protocol on verified reference materials sourced from the SPS-WW2 batch. The study of the certified reference materials (CRMs) involved the application of Student’s t-test, as indicated by the findings presented in Table 2. Based on the obtained t-test values of 0.4818 for Co^2+^, 0.2117 for Cr^3+^, and 0.3005 for Ni^2+^, which are all below the critical value of 4.303 at a 95% confidence level, it can be inferred that there is no statistically significant difference between the concentrations determined using the proposed methodology and the certified values of the CRMs. The study’s findings indicate that the proposed method is appropriate for analyzing real samples.

### 3.6. Comparison of the proposed method with other reported methods

This study aimed to conduct a comparative analysis between the suggested method and many previously published methodologies [28–31] for the extraction and preconcentration of the studied metal ions, as demonstrated in Table 3. The results showed that the new method exhibits equivalent or superior linearity, LOD, and precision compared to previously published methods. The LOD achieved in this investigation demonstrates similarity to that observed in other methodologies. In comparison to other preconcentration techniques, the preconcentration factor exhibited by Fe_3_O_4_@coPPy-PTH/MIS-FAAS surpassed that of certain previously documented methods. The suggested methodology offers numerous advantageous characteristics, particularly in terms of solvent utilization, rapid extraction, and the capacity to extract multiple analytes concurrently. Furthermore, the approach exhibits satisfactory analytical performance and sensitivity. Additionally, the proposed methodology does not require the use of a chelating agent or organic solvent, and it exhibits rapidity and selectivity. The feasibility of utilizing the Fe_3_O_4_@coPPy-PTH/MIS-FAAS technique for the determination of Co(II), Cr(III), and Ni(II) in beer and wine matrices was demonstrated by the analytical performance characteristics.

### 3.7. Application of the proposed method in the analysis of real samples

The proposed method was employed with synthesized materials for the extraction and determination of Co(II), Cr(III), and Ni(II) in different beer and wine samples. To evaluate the matrix effect on the performance of the method and to validate its accuracy and applicability, real sample analysis was done using beer and different wines. The sample preparations and extraction techniques were conducted for the appropriate sections. Genuine samples were augmented with a suitable quantity of analytes and the following recoveries were determined by evaluating the quantities of the added analytes. The relative recovery (RR) was calculated with the following equation:


(1) $$\text{RR}\%=\frac{{\text{C}}_{\text{found}}-{\text{C}}_{\text{real}}}{{\text{C}}_{\text{added}}}\times 100$$

Here, C_found_, C_real_, and C_added_ represent the concentrations of the analyte found after analysis, the concentration of the analyte in the real sample, and the concentration of a known quantity of the standard that was introduced into the actual sample, respectively. The calculated recoveries for the spiked samples fell within an acceptable range of 88.6% to 103.6%. Additionally, the RSDs for these recoveries varied from 0.5% to 3.3%. These results confirm the accuracy of the detected analyte quantities in nonspiked real samples. Table 4 illustrates the concordance between the triplicate analyses of each sample conducted using the recommended methodology and the spiking quantities. The proposed methodology demonstrates satisfactory recoveries and precision in the analysis of metal ions in real samples.

## 4. Conclusion

The present study involved the synthesis of Fe_3_O_4_@coPPy-PTH nanocomposite, which was subsequently employed as a highly efficient adsorbent for the simultaneous extraction of Co(II), Cr(III), and Ni(II) metal ions followed by the measurement of metal ions by MIS-FAAS. The presence of Fe_3_O_4_ gives the adsorbent magnetic properties, making it easy to retrieve and separate the relevant ions using an external magnetic field. This simplifies the extraction procedure and diminishes the necessity for significant filtration or centrifugation. The coPPy-PTH coating provides excellent selectivity and strong attraction for metal ions, guaranteeing effective extraction even when other compounds are present. Additionally, the produced nanocomposite’s substantial specific surface area facilitated its utilization and accelerated the analytical method. The system demonstrated exceptional selectivity, remarkable recovery rates, accurate readings, and a robust PF. The proposed method obviated the necessity for chelating agents or organic solvents, commonly employed to sequester and extract metal ions. While this strategy has advantages, it may also have certain disadvantages. Occasionally, the MSPE approach lacks the ability to fully separate the magnetic adsorbent from the sample matrix being used. Both contamination and deterioration in the quality of the extracted analytes may result from this. In addition, the process of creating and altering magnetic adsorbents can be both arduous and expensive. These adsorbents necessitate high-quality components and can be difficult to manage during the manufacturing process.

The future prospects of improving the fabrication and manipulation of Fe_3_O_4_@coPPy-PTH nanocomposites have the potential to boost their ability to specifically remove and absorb hazardous substances, making them highly suitable for environmental restoration purposes. Ongoing efforts are being made to enhance the cost-effectiveness and flexibility of Fe_3_O_4_@coPPy-PTH nanocomposites through research and development. As a result, they are likely to be widely used in analytical and industrial environments, especially for the elimination of small contaminants.

## Figures and Tables

**Table 1:** Analytical features of the applied MSPE method using Fe_3_O_4_@coPPy-PTH nanocomposite as an adsorbent.

Analyte	Linearity (μg L^−1^)		LOD (μg L^−1^)	ER (%)	Precision a
	
	LDR (μg L^−1^)	R^2^			(RSD%, n = 5)
Co(II)	0.0–10.0	0.9992	0.17	88.2	1.5
Cr(III)	0.0–10.0	0.9781	0.22	94.6	1.2
Ni(II)	0.0–10.0	0.9957	0.23	93.6	3.0

a Calculated based on the extraction of 10 μg L^−1^ of each analyte.

R^2^: Coefficient of determination; ER, extraction recovery; RSD, relative standard deviation.

**Table 2:** Analysis of CRMs using the proposed procedure. Conditions: volume of SPS-WW2 batch, 16–25.01 mL; n = 3.

Parameter	SPS-WW2 batch 16 (μg L^−1^)		

	Co	Cr	Ni
Certified concentration	0.3 ± 0.02	1.00 ± 0.05	5.00 ± 0.03
Found concentration	0.29 ± 0.01	0.95 ± 0.03	4.96 ± 0.05
Relative error, %	3.3	5.0	0.8
RSD, %	3.4	3.2	1.0
t_test_	0.4818	0.2117	0.3005
Significance a	Statistically identical	Statistically identical	Statistically identical

a Significance of t-test (n = 3) at the 95% confidence level, t_critical2_ = 4.303.

**Table 3:** Comparison of the figures of merit of the proposed method with other reported methods for extraction and determination of Co(II), Cr(III), and Ni(II).

Method	Analyte	Matrix	LDR (μg L^−1^)	R^2^	LOD (μg L^−1^)	RSD%	Ref.

NH-IS-SPE-μS-FAAS	Co(II)	Water, cow liver, spinach, meat, blood	10.0–200	0.9994	2.95	3.5	[28]
	Cr(III)		10.0–200	0.9990	3.10	3.2	
	Ni(II)		10.0–250	0.9990	2.74	3.5	

MSPE-FI-ICP-OES	Co(II)	Tap water, mineral water, river water	0.50–100	0.9980	0.20	6.8	[29]
	Cr(III)		0.20–100	0.9986	0.10	6.1	

d-CPE-ICP-OES	Co(II)	Water samples	-	0.9917	0.01	4.2	[30]
	Ni(II)		-	0.9982	0.01	5.6	

DTO-SPE-FAAS	Co(II)	River water, spring water, soil, blood, vegetable	18.0–900	-	0.80	0.85	[31]
	Ni(II)		17.0–850	-	0.75	0.90	

Fe_3_O_4_@coPPy-PTH-MIS-FAAS	Co(II)	Red wine, red wine, rosé wine, white wine, beer, mineral water, drinking water	0.0–10.0	0.9992	0.17	1.5	This work
	Cr(III)		0.0–10.0	0.9781	0.22	1.2	
	Ni(II)		0.0–10.0	0.9957	0.23	3.0	

**Table 4:** Determination of studied analytes in real samples using Fe_3_O_4_@coPPy-PTH nanocomposite as an adsorbent followed by the MSPE method.

Sample		Co(II)	Cr(III)	Ni(II)

Red wine (Syrah)	Found^a^ (Added^b^)	1.13 ± 0.01 (1.25)	1.30 ± 0.01 (1.25)	
	RR^c^ %	90.2	103.6	1.16 ± 0.03 (1.25)
	RSD (n = 3)	1.2	1.1	92.5 2.4

Red wine (Merlot)	Found^a^ (Added^b^)	1.15 ± 0.04 (1.25)	1.28 ± 0.01 (1.25)	1.20 ± 0.03 (1.25)
	RR^c^ %	92.0	102.8	96.0
	RSD (n = 3)	3.3	0.6	2.4

Rosé wine	Found^a^ (Added^b^)	1.27 ± 0.01 (1.25)	1.25 ± 0.01 (1.25)	1.15 ± 0.04 (1.25)
	RR^c^ %	101.4	100.0	92.1
	RSD (n = 3)	0.6	0.5	3.0

White wine	Found^a^ (Added^b^)	1.11 ± 0.02 (1.25)	1.20 ± 0.03 (1.25)	1.18 ± 0.03 (1.25)
	RR^c^ %	88.6	96.2	94.5
	RSD (n = 3)	1.7	2.2	2.8

Beer	Found^a^ (Added^b^)	1.17 ± 0.03 (1.25)	1.28 ± 0.03 (1.25)	1.28 ± 0.02 (1.25)
	RR^c^ %	93.5	102.4	102.7 1.8
	RSD (n = 3)	2.9	2.7	

Mineral water	Found^a^ (Added^b^)	1.21 ± 0.01 (1.25)	1.20 ± 0.02 (1.25)	1.28 ± 0.03 (1.25)
	RR^c^ %	97.0	95.6	102.5
	RSD (n = 3)	0.8	1.7	2.3

Drinking water	Found^a^ (Added^b^)	1.23 ± 0.01 (1.25)	1.22 ± 0.02 (1.25)	1.25 ± 0.03 (1.25)
	RR^c^ %	98.7	97.4	99.9
	RSD (n = 3)	0.9	1.9	2.3

a Concentration observed after spiking (μg L^−1^).

b Spiked concentration (μg L^−1^).

c Relative recovery.
